# Complete mitochondrial genome of *Mycalesis intermedia* (Lepidoptera: Nymphalidae)

**DOI:** 10.1080/23802359.2020.1714491

**Published:** 2020-01-21

**Authors:** Yu-Peng Wu, Jun-Jiao Lu, Jing Yang, Ju-Ping Wang, Tian-Wen Cao, Ren-Jun Fan

**Affiliations:** a College of Environment and Safety, Taiyuan University of Science and Technology, Taiyuan, China;; b Institute of Plant Protection, Shanxi Academy of Agricultural Sciences, Taiyuan, China

**Keywords:** *Mycalesis intermedia*, complete mitogenome, Lepidoptera

## Abstract

The *Mycalesis intermedia* belongs to Nymphalidae in Lepidoptera. We described the complete mitogenome of *M. intermedia*, which is typical circular duplex molecules and 15,386 bp in length, containing the standard metazoan set of 13 protein-coding genes, 22 transfer RNA genes, 2 ribosomal RNA genes, and an A + T-rich region with macro-repeat sequences. All the inferred tRNA secondary structures show the common cloverleaf pattern, with the exception of *trnS1(AGN)* which lacks the DHU arm. The *M. intermedia* mitochondrial genome has the same gene order with other lepidopterans.

The *Mycalesis intermedia* Moore [1892] is a forestry pest, and widely distributed in world (Mayekar and Kodandaramaiah 2017). In this study, We sequenced and described the complete mitogenome of *M. intermedia* to provide useful genetic information for species identification and phylogenetic analysis (Lu et al. 2013). The specimens of *M. intermedia* a were collected by light trapping in Taiyuan, China (37.833393, 112.666114) in July 2018, some of these specimens were immediately frozen in −80 °C on board for mitogenome analysis, and others were preserved by spreading wings in the Herbarium of Institute of Plant Protection, Shanxi Academy of Agricultural Sciences, and their numbers are TYKD20190601-20190604.

The complete mitogenome sequence of *M. intermedia* is 15386 bp (GenBank accession number: MN610565). As with other insect mitogenomes, the major strand encodes a larger number of genes (9 PCGs and 14 tRNAs) than the minor strand (4 PCGs, 8 tRNAs and 2 rRNA genes). A large non-coding, A + T-rich region in insects is present between *rrnS* and *trnM* . The mitochondrial gene order of *M. intermedia* is identical to those of all other sequenced lepidopterans (Hu et al. 2010).

Twelve PCGs have the usual start codon ATN, but the *cox1* gene commences with exceptional codon CGA, which was found in another insects as the initial codon (Wu, Zhao, Su, Luo, et al. 2016). Twelve PCGs have the common stop codon TAA, except for *nad4* have the in-complete stop codon T.

The *M. intermedia* mitogenome is biased toward A + T (80.95%). The overall base composition is 39.24% A, 41.62% T, 7.32% G and 11.82% C. The 22 tRNA genes ranged from 60 to 70 nucleotides. Fourteen tRNAs are coded on the J-strand and others on the N-strand. Complete cloverleaf secondary structures could be inferred for 21 of the 22 tRNAs. The secondary structure of *trnS1(AGN)* is incomplete, lacking the DHU arm. The *rrnL* gene (1375 bp) is located between *trnL(CUN)* and *trnV*, and the *rrnS* (770 bp) between *trnV* and the A + T-rich region.

The mitogenome includes 14 overlapping regions ranging from 2 bp to 55 bp and 17 intergenic spacers ranging from 1 bp to 35 bp, with the exception of the A + T-rich region (the largest non-coding region). The A + T-rich region (512 bp) is known for regulating transcription and replication of the mitogenome (Wu, Zhao, Su, He, et al. 2016). There is a motif ATAGA in downstream of *rrnS* followed by an 19 bp, and after 81 bp interval there is another 18 bp Poly-T stretch. The phylogenetic relationships were constructed based on the complete mitochondrial genomes of butterflies represented eight Family of Lepidoptera (Figure 1). The sequences were aligned with MAFFT v7.2 software (Katoh and Standley 2013) and the evolutionary analyses were conducted with RAxML v8.2.10 (Stamatakis 2014) on the CIPRES Science Gateway (Miller et al. 2010). The GTRGAMMA model with ‘Let RAxML halt bootstrapping automatically’ was used. The phylogenetic tree was visualized using FigTree v1.4.4 (FigTree 2018).

**Figure 1.**
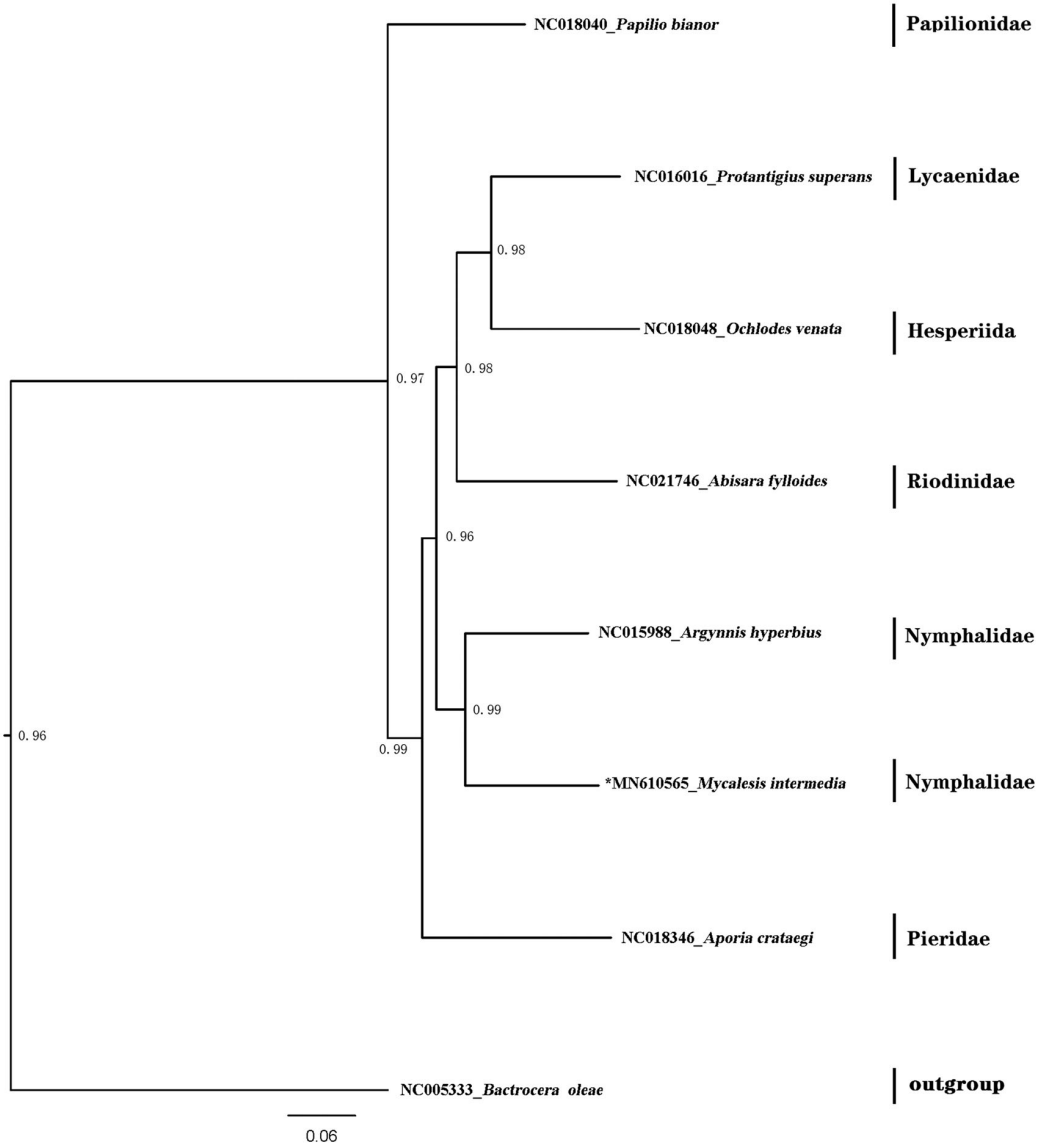
Maximum-likelihood tree of evolutionary relationships *Mycalesis intermedia* based on the complete mitogenomes of 8 Lepidopteran butterflies. *M. intermedia* and *Argynnis hyperbius* are clustered into a clade Nymphalidae.

## References

[CIT0001] FigTree - molecular evolution, phylogenetics and epidemiology. 2018. [accessed 2018 Nov 26]. http://tree.bio.ed.ac.uk/software/figtree/.

[CIT0002] Hu J, Zhang DX, Hao JS, Huang DY, Cameron S, Zhu CD. 2010. The complete mitochondrial genome of the yellow coaster, *Acraea issoria* (Lepidoptera: Nymphalidae: Heliconiinae: Acraeini): sequence, gene organization and a unique tRNA translocation event. Mol Biol Rep. 37(7):3431–3438. 20091125 10.1007/s11033-009-9934-3

[CIT0003] Katoh K, Standley DM. 2013. MAFFT multiple sequence alignment software version 7: improvements in performance and usability. Mol Biol Evol. 30(4):772–780. 23329690 10.1093/molbev/mst010 PMC3603318

[CIT0004] Lu HF, Su TJ, Luo AR, Zhu CD, Wu CS. 2013. Characterization of the complete mitochondrion genome of diurnal moth *Amata emma* (Butler) (Lepidoptera: Erebidae) and its phylogenetic implications. PloS One. 8(9):e72410. 24069145 10.1371/journal.pone.0072410 PMC3771990

[CIT0005] Mayekar HV, Kodandaramaiah U. 2017. Pupal colour plasticity in a tropical butterfly, *Mycalesis mineus* (Nymphalidae: Satyrinae). PLoS One. 12(2):e0171482. 28158254 10.1371/journal.pone.0171482 PMC5291534

[CIT0006] Miller MA, Pfeiffer W, Schwartz T. 2010. Creating the CIPRES science gateway for inference of large phylogenetic trees. Proceedings of the Gateway Computing Environments Workshop (GCE); Nov 14; New Orleans, Louisiana: Institute of Electrical and Electronics Engineers (IEEE); p. 1–8.

[CIT0007] Stamatakis A. 2014. RAxML version 8: a tool for phylogenetic analysis and post-analysis of large phylogenies. Bioinformatics. 30(9):1312–1313. 24451623 10.1093/bioinformatics/btu033 PMC3998144

[CIT0008] Wu YP, Zhao JL, Su TJ, Luo AR, Zhu CD. 2016. The complete mitochondrial genome of *Choristoneura longicellana* (Lepidoptera: Tortricidae) and phylogenetic analysis of Lepidoptera. Gene. 591(1):161–176. 27390085 10.1016/j.gene.2016.07.003

[CIT0009] Wu YP, Zhao JL, Su TJ, He QS, Xie JL, Zhu CD. 2016. The complete mitochondrial genome of *Carposina sasakii* (Lepidoptera: Carposinidae). Mitochondrial DNA. 27(2):1432–1434. 25185454 10.3109/19401736.2014.953079

